# 

**Published:** 1887-11-19

**Authors:** 


No. 60, Vol. III—November 19th, 1887.

Registered at the General Post Office as a Newspaper.]

### THE HOSPITAL

Per Annum, 6s. 6d., post free.

Monthly Parts, 6d.; post free, 7½d.

Ditto per Ann., 7s. 6d, post free.

